# Chronodentistry: the role & potential of molecular clocks in oral medicine

**DOI:** 10.1186/s12903-019-0720-x

**Published:** 2019-02-13

**Authors:** Klara Janjić, Hermann Agis

**Affiliations:** 1 Department of Conservative Dentistry and Periodontology, University Clinic of Dentistry, Medical University of Vienna, Sensengasse 2a, 1090 Vienna, Austria; 2 Austrian Cluster for Tissue Regeneration, Vienna, Austria

**Keywords:** Circadian clock, Molecular clock, Dentistry, Chronopharmacology, Oral surgery, Conservative dentistry, Endodontology, Periodontology

## Abstract

Molecular clocks help organisms to adapt important physiological functions to periodically changing conditions in the environment. These include the adaption of the 24 h sleep-wake rhythm to changes of day and night. The circadian clock is known to act as a key regulator in processes of health and disease in different organs. The knowledge on the circadian clock led to the development of chronopharmacology and chronotherapy. These fields aim to investigate how efficiency of medication and therapies can be improved based on circadian clock mechanisms. In this review we aim to highlight the role of the circadian clock in oral tissues and its potential in the different fields of dentistry including oral and maxillofacial surgery, restorative dentistry, endodontics, periodontics and orthodontics to trigger the evolving field of chronodentistry.

## Background

**Fig. 1:**
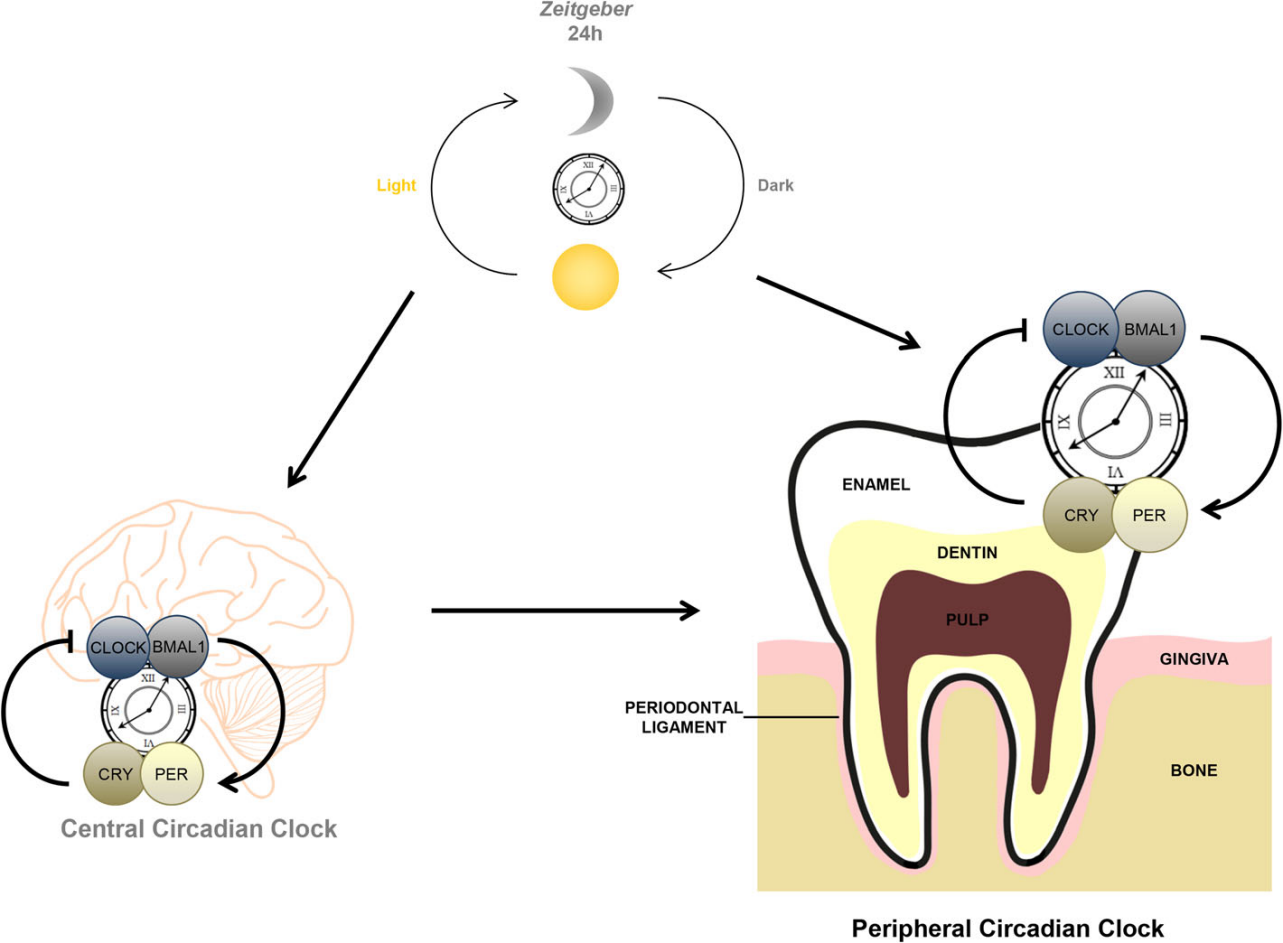
The circadian clock mechanism. Daily alternation between light/dark periods during days/nights are stimuli from the environment (*zeitgeber* [official technical term]) that entrain 24 h circadian rhythms. The stimuli are received by the central circadian clock in suprachiasmatic nucleus of the brain, regulating the transcriptional-translational feedback loop between the core components of the circadian clock: circadian locomotor output cycles kaput (CLOCK), aryl hydrocarbon receptor nuclear translocator-like (BMAL1), cryptochrome (CRY) and period (PER). Peripheral circadian clocks in different oral tissues receive signals from the central circadian clock or directly from the *zeitgeber* providing time keeping of physiological functions. Adapted from [91]

As part of evolution, the adaption to environmental changes contributes fundamentally to prevail in natural selection. Hence this ability is an important feature of a healthy organism. Teeth play a major role in satisfying the basic human need of food uptake. Therefore, throughout evolution, dental patterning, morphology and genetics of tooth development had to adjust in a way to match food supply and requirements of the body [1]. At molecular levels, this ability to synchronize to changing conditions in the environment is driven by a machinery called the molecular clocks. Depending on the cycle length of respective biological rhythms, different types of molecular clocks have been defined: the circadian clock (24 h periods); adapting to daily changes, the circalunar clock (29.5 d periods); adapting to moon phases and the circannual clock (365 d periods); adapting to seasonal changes [2]. Among these, the circadian clock is the most studied one. A variety of factors as light, food, body temperature or cellular redox status were identified as input, also termed *zeitgeber*, to set the pace for circadian clocks [3]. Depending on these inputs, the circadian clock governs synchronization of various physiological processes as an output to the environmental changes. The inputs are transmitted to the central circadian clock in the brain [4] that controls cell-autonomous peripheral circadian clocks in different organs [3]. Until now, the presence of peripheral circadian clocks has been shown in almost all human organs, including the cardiovascular system [5], the respiratory system [6], the musculoskeletal system [7, 8], skin [9] and the digestive system [10], where the circadian clock is responsible for conducting physiological functions and behavior. First attempts to discover the circadian clock in dental tissues focused on tooth development and only recently evidence was raised that also oral tissues in adults contain a peripheral clock [11] (Fig. 1). To be able to understand the role of the circadian clock system thoroughly, not only presence and function have to be investigated, but also demonstrating consequences of dysregulation is of importance. Interruption of the circadian clock mechanisms gave hints to implications in various diseases as diabetes [12, 13], osteoporosis [14], cancer [15] and immune-allergic diseases [16]. Finding the links between the circadian clock and diseases is of particular importance these days, since modern living comes along with numerous factors, which disturb circadian rhythms: artificial light of different sources being available at all times, irregular food uptake, shift work and so on.

**Fig. 2:**
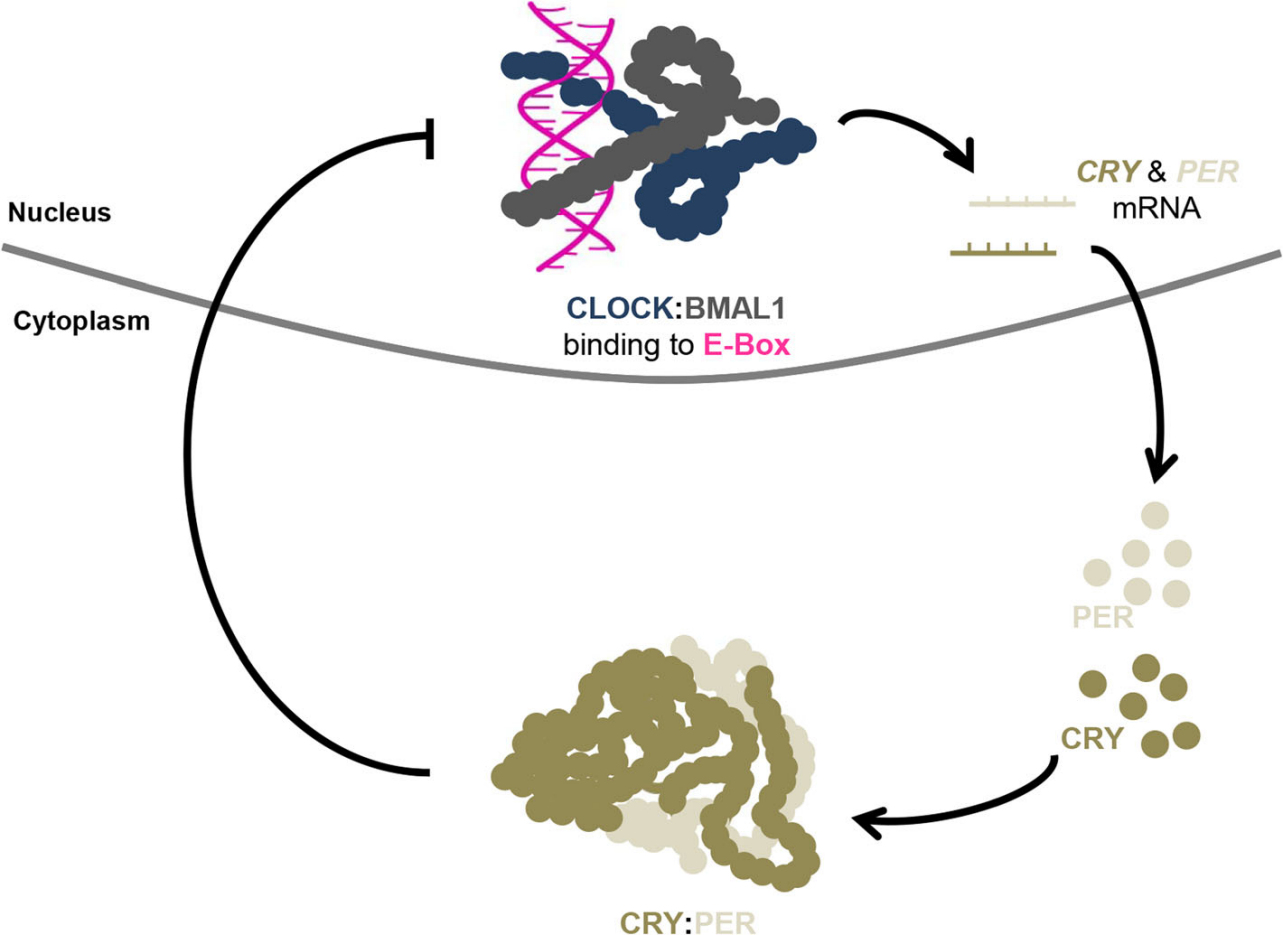
The transcriptional-translational feedback loop of the circadian clock. Circadian locomotor output cycles kaput (CLOCK) and aryl hydrocarbon receptor nuclear translocator-like (BMAL1) dimerize in the cell nucleus (CLOCK:BMAL1) to act as transcription factors when binding to E-box elements in the promoter regions of cryptochrome (*CRY*) and period (*PER*). Produced *CRY* and *PER* mRNA is translated in the cytoplasm to CRY and PER proteins. CRY and PER accumulate and form a dimer (CRY:PER) that inhibits CLOCK:BMAL1 activity. Adapted from [92]

To gain deeper insights into the processes where the circadian clock is implied in physiology or pathology, attempts are made to find underlying molecular mechanisms. The circadian clock mechanism is based on a transcriptional-translational feedback loop which works cell-autonomously. This means that cells do not depend on the connection to a central circadian clock to display a working circadian mechanism [17]. Circadian locomotor output cycles kaput (Clock), aryl hydrocarbon receptor nuclear translocator-like (Arntl or Bmal1), cryptochrome 1–2 (Cry1–2) and period 1–3 (Per1–3) build the key components of the mammalian clockwork and interact in a transcriptional-translational feedback mechanism. Daylight as input stimulates coupling of CLOCK and BMAL1, initiating transcription of *CRY* and *PER*. Upon a certain level of mRNA, their protein products CRY and PER dimerize and inhibit CLOCK:BMAL1 complexes in the nucleus, thereby inhibiting their own transcription. With the light of the night the dimer gets degraded, allowing for activation of CLOCK and BMAL1 in a new transcription cycle [18] (Fig. 2). Based on this molecular mechanism, the circadian timing system (CTS) regulates a number of physiological functions when the central circadian clock in the brain receives light or dark impulses and transmits them to peripheral clocks as those in oral tissues in a 24 h rhythm. Certain types of lifestyle which deviate strongly from this daily rhythmicity can cause poor coordination of peripheral clocks, ultimately leading to a dysfunctional clock. For example, shift work is associated with an increased incidence of oral health problems [19] and circadian production of melatonin, a sleep-related hormone, seems to be correlated with tooth development [20]. A functional CTS is thus relevant for oral health. Further, knowing the functional CTS can be used for directed therapies by minding its timings to maximize efficacy while reducing adverse effects.

Chronopharmacology and chronotherapy are fields that make use of the knowledge on chronobiology to improve the outcome of medication and therapies [21]. As it was found that pharmacokinetics and pharmacodynamics follow daily rhythms [22] it seems likely that optimizing timing of drug administration to circadian oscillations could increase effectiveness and efficiency of respective applications. While classical pharmacology focuses on dose finding and exposure time studies, a review on chronopharmacology from 2016 [21] reports that so far over 300 medications have been tested for effects of different dosing-timings. Tested effects were not limited to increased outcome, but also included investigations of decreased side effects and general medication safety. A precise timing of drug use could also relieve patient’s metabolism, reduce costs and required appointments at the dentist’s, altogether improving daily routines. Biological rhythms during the course of a day differ from one person to another. The chronotype of a person determines preferential times of a day for biological rhythms and can be easily determined by a chronotype questionnaire. Chronotypes correlate with circadian rhythms. Thus, individuals with a certain chronotype are associated with e.g. metabolic disorders as diabetes [23] stronger than individuals with a different chronotype. Therefore, analysis of an individual’s chronotype could be helpful for diagnosis and treatment.

Chronodentistry opens great potentials for advances in dental applications. The first steps have already been made, but there is still a lot left to discover and develop. Within the scope of this review we summarized current knowledge on the circadian clock in oral tissues with the aim to raise awareness for findings that are relevant for the main fields of dental practice.

### Oral & maxillofacial surgery

#### Biological aspects

The dysregulation of angiogenesis, growth, cell proliferation and cell death are regarded as hallmarks of cancer [24]. As the circadian clock is implied in the regulation of blood vessel formation [25], the cell cycle and growth rates of cells [26], it raises interest as target in the field of tumor biology and cancer treatment. In oral cancer, the different PER stand out as components with an impact on many aspects of carcinogenesis as increased downregulation of PER1 correlates with tumor progression [27], diurnal rhythms of PER1 are correlated with carcinogenesis [28], a tumor suppressor role is suggested for PER2 [29] and decreased levels of PER1 are associated with later stages of cancer and lymph node metastasis [30]. Further, modulations of PER1, PER2, DEC1, DEC2, CRY1, CRY2, NPAS2, PER3, TIM, RORα and REV-ERBα were associated with cell proliferation, apoptosis and cell cycle progression in oral squamous cell carcinoma cells [31–34], implying a potential role in tumorigenesis. Beside PER, also BMAL1 has been suggested to play a role in head and neck squamous cell carcinoma, being dependent on the oxidation of the tumor suppressor gene PTEN over the mTOR pathway [35]. Also in bone a function of Bmal1 was discovered in mandibles with juvenile skeletal mandibular hypoplasia. Apparently, Bmal1 dysfunction leads to this pathology over a matrix metalloproteinase 3 pathway [36]. Bmal1 and other core clock components play further roles during bone remodeling [37, 38], a process that contributes to the outcome of dental implants as well as pre-implantation procedures like bone augmentations. Studying the role of the circadian clock in healthy and diseased bone could be a key to better understanding of bone pathologies relevant in the maxillomandibular field.

#### Clinical aspects

In implantology, biomaterials containing titanium are frequently used for dental implants. Recently it was found that Per1 in bone marrow stromal cells is downregulated due to titanium-based biomaterials, which could be relevant for osseointegration [39]. Patients diagnosed with a head and neck squamous cell carcinoma showed a significant dysregulation of circadian clock core components BMAL1, CRY1, CRY2, PER1, PER2, PER3 and CASEIN KINASE 1ε (CK1ε), depending on the stage of tumor development as advanced cancer stages correlated with downregulation of BMAL1, CRY2 and PER3 [35]. Downregulation of PER3 and upregulation of TIMELESS (TIM) were characteristic for larger tumors, downregulation of PER3 for deeper tumor invasion and downregulation of PER1 and PER3 was associated with poor patient survival [40]. Interestingly, patients of head and neck squamous cell carcinoma also had downregulated circadian clock genes in their peripheral blood leukocytes before surgery while after surgery CLOCK and PER1 recovered in those patients with a good prognosis, but not in those who died within one year after surgery [41]. In healthy individuals, tumor suppressor genes and oncogenes were identified as clock-controlled genes in human oral mucosa [42]. Using the knowledge of circadian clock mechanisms for oral and maxillofacial surgery enables development of therapeutic strategies with the possibility to interact at more complex levels than drugs or therapies that influence only one specific target. Especially for oral cancer patients a directed use of the circadian clock could be promising [43]. Disrupted cell cycle control is a characteristic in many cancers. An association between cell cycle phases in human oral mucosa cells and clock gene expression has been found [44], supporting chronotherapeutic approaches for cancer treatment. Chemotherapeutics cause severe adverse effects, which could be reduced if they could be applied as effectively at lower doses in a determined time frame. Chronochemotherapy is a concept that is based on this idea. Several studies showed that chronochemotherapy treatment against oral squamous cell carcinoma [45] and nasopharyngeal carcinoma [46, 47] yielded reduced incidence of adverse effects, increase of treatment tolerance, improved survival time and reduction of stomatitis. Also chronoradiotherapy shows similar promising results in nasopharyngeal carcinoma [48].

### Restorative dentistry

#### Biological aspects

Enamel is one of the mineralized tissues of our body, forming during amelogenesis. Studying this process is of particular interest since diseased or traumatized enamel is not capable of self-repair. The circadian clock is involved in bone remodeling [23, 49], thus it regulates the homeostasis in another mineralized tissue. Therefore, it has been hypothesized that the circadian clock could have an influence during enamel formation. Production of amelogenin (*Amelx*) and kallikrein-related peptidase 4 (*Klk4*), markers for the secretory and the maturation phase of amelogenesis, respectively, is proposed to be stimulated by *Bmal1* in a rat cell line [50]. *Amelx* is decreased during dark periods whereas markers relevant for tooth development such as lysosomal associated membrane protein 1 (*Lamp1*) for matrix endocytosis, sodium-bicarbonate cotransporter *(Slc4a4)* for bicarbonate transport and carbonic anhydrase 2 *(Car2)* for bicarbonate production were increased during dark periods in mice [51]. Taking a closer look at the different stages of tooth development, a study found *Bmal1*, *Clock*, *Per1* and *Per2* to be produced in whole murine tooth extracts one day after birth. During bell stage, PER1 showed the strongest expression, followed by CLOCK and PER2 in terms of intensity and also BMAL1 showed weak but clear production in ameloblasts. Four days after birth BMAL1 was upregulated in nuclei of ameloblasts and CLOCK, PER1 and PER2 continued to be strongly produced in ameloblast nuclei. [52] Some publications also suggest a correlation between circadian periodicity, cross-striations and incremental lines in histological tooth sections [53–55]. Further investigations on the oscillation courses revealed that *Bmal1* and *Per2* oscillate in antiphase to each other in synchronized murine ameloblasts [51]. The exact mechanisms and the interplay between clock genes and clock-controlled genes are yet unrevealed. First attempts to clarify underlying mechanisms found that runt-related transcription factor 2 (*Runx2*) overexpression in a rat ameloblast cell line downregulated *Amelx* and enamelin (*Enam*) at steady state whereas distal-less homeobox 3 (*Dlx3*) overexpression upregulated mRNA production of same genes. Although used cells were synchronized, the involvement of clock genes has not been demonstrated yet [56].

Dentin is another hard tissue of the tooth. During tooth development activities of clock components in ameloblasts are paralleled by odontoblasts during bell stage and four days after birth, as mentioned above. Interestingly, on day 21 after birth, clock proteins were downregulated in odontoblasts of the crown analogue side whereas the root analogue side continued to express clock proteins [52]. Dentin formation is also characterized by incremental lines. For these incremental lines a circadian pattern was observed in mammals [57]. In odontoblasts, a circadian rhythm in collagen production and secretion was revealed which could contribute to the rhythmicity of incremental lines in dentin [57]. This finding was supported by another study, suggesting that incremental lines are regulated by the suprachiasmatic nucleus, the location of the central circadian clock [58].

#### Clinical aspects

Restorative dentistry mainly relies on dental materials for fillings when it comes to the repair of enamel defects. Deeper knowledge of the role of circadian clocks in dental hard tissue formation could act as cue to develop new therapeutic strategies. Genetic polymorphisms in the circadian clock system could be a source for individual alterations in enamel morphology, thickness and hardness [50]. If so, this could be a target for new therapies with regenerative potential for dental hard tissue instead of only providing repair with unforeseeable durability.

### Endodontics

#### Biological aspects

In the course of tooth development the dental pulp evolves from the mesenchymal germ layer, passing different embryonic stages. During this process several molecular pathways as the transforming growth factor β, bone morphogenetic protein, tumor necrosis factor, sonic hedgehog, fibroblast growth factor and the wnt signaling pathway were suggested to communicate within the different developmental stages [59]. As the circadian clock was claimed to play a role in cell differentiation [60], its role during development was of interest. A study in murine dental pulp cells reports findings of only sporadic CLOCK, BMAL1, PER1 and PER2 production with variable levels, observed in three different embryonic stages. On day 4 after birth named proteins were still produced but at lower levels except for PER1 which showed higher production intensities. 21 days after birth none of the proteins could be detected anymore [52]. One portion of the heterogeneous cell population in the dental pulp tissue is represented by dental pulp stem cells which are currently evaluated as source for a number of regenerative approaches [61]. Dental pulp stem cells show oscillatory production of *BMAL1*, *PER2* and *REV-ERBα* after synchronization by mechanical stretching while chemical synchronization did not yield comparable results [62].

#### Clinical aspects

In endodontics, root canal treatment is part of the routine therapeutic interventions and its success depends among other things on the in-depth elimination of infectious microorganisms. Photodynamic therapy was proposed for root canal system disinfection exerting its effects by production of reactive oxygen species (ROS), which is toxic for tumor cells, bacteria and fungi [63]. Since light is the main *zeitgeber* for the circadian clock, photodynamic treatment could also modulate the circadian clock feedback mechanism. In addition it has been shown that ROS stress re-sets the circadian clock, leading to a stimulation of cell survival [63]. Studying circadian clock mechanisms in this context might help to improve the outcome of photodynamic therapies. One approach could be to adapt the wave length, therapy timing and exposure time to the specific chronotype of a patient. Patients suffering from inflamed or traumatized dental pulps are particularly sensitive in the affected region. However, until we do not have data on the role of the circadian clock in these settings, all of this remains speculation.

Depending on the redox status in cells, zinc influences formation of the CRY1:PER2 complex [64]. Zinc oxide eugenol is used as material for pulp capping. It would be of interest if other pulp capping materials modulate circadian clock components in the dental pulp, but currently there is no literature on this topic. It is known that PER2 and PER3 are downregulated in dental pulps from carious teeth [65]. Future research is required to find out which effects pulp capping has on the circadian clock in the dental pulp and how this knowledge can be adopted in treatment. Further it is unknown if the chronotype is correlated to success of endodontic treatments like pulp capping. In elderly diabetic and hypertensive patients an altered rhythm of pulp sensibility has been determined, but even in healthy individuals pulp sensibility seems to follow diurnal rhythms [66]. Following these findings, appointments for endodontic treatments could be adapted according to individual pulp sensibility rhythms or chronotypes. Pain sensation and analgesic treatment in oral regions has been suggested to correlate with circadian phases a long time ago [67–70]. Further studies will be required to further investigate the influence of the circadian clock of pain perception in the tooth for establishing feasible treatment protocols. However, this would be an easy way to improve patient comfort during treatments.

### Periodontics

#### Biological aspects

Periodontal soft tissue consists of gingiva and periodontal ligament. It is known that the two positive transcriptional-translational regulators CLOCK and BMAL1 exert their function by binding to E-boxes with a CACGTG sequence in the promoter region of *PER1–2*, *CRY1–2* and differentiated embryo-chondrocyte 1–2 (*DEC1–2*). Human *SMAD3* and *SNAIL* have the same promoter sequence in common and were found to also show circadian expression in human gingival fibroblasts. However, only the promoter activity of *SMAD3*, but not *SNAIL* was upregulated by CLOCK:BMAL1. Therefore, the promoter sequence can be a good hint to a clock-controlled gene, but is not a guarantee. Also hypoxia-inducible factor-1 (*HIF-1*) shares the same promoter sequence, but did not show circadian oscillation in gingival fibroblasts. VEGF, which is regulated by HIF, does on the other hand show circadian expression [71]. Generally, its expression is enhanced under hypoxic conditions. Hypoxia or hypoxia mimetic agents are also known to re-set the cell cycle and are therefore used for cell synchronization. When looking at the effects of hypoxia on the circadian clock components in gingival fibroblasts and periodontal ligament fibroblasts it has been shown that *CLOCK*, *CRY1–2* and *PER3* are downregulated at mRNA levels [11], but not at protein levels (unpublished observation). Further studies will be required to find the mechanisms behind this effect. Gingival fibroblasts also play a role in diseased conditions, for example when they form part of the microenvironment of oral cancer cells. Interestingly, co-culture of human gingival fibroblasts and oral cancer cells alters clock gene oscillations, pointing to a role in oral cancer development (Furudate K et al. 2016).

#### Clinical aspects

Easy achievable parameters for diagnosis of periodontitis as well as successful control after treatment are desirable for each periodontist. For example, increased interleukin (IL)-1β levels in crevicular fluid are considered as marker for gingivitis or periodontitis while decreased levels correlate with successful therapy [72]. It has been found now that IL-1β underlies level variations during a day in individuals without periodontal inflammation [72]. Here, clinical assessment could be more precise when deciding on the best moment to test for markers. Also the periodontal indices full-mouth bleeding score (FMBS), full-mouth plaque score (FMPS), periodontal screening and recording (PSR) and periodontal risk assessment (PRA) were suggested to underlie daily variations in healthy subjects [73]. Other candidate markers used in clinics to monitor health status of the periodontium remain to be assessed in their daily detection behavior. Alveolar bone loss is regarded as a major challenge in periodontitis [74]. An influence of the circadian clock on bone resorption activity was shown in osteoblasts and osteoclasts [75]. It was suggested that circadian rhythms are transmitted glucocorticoid-mediated from the central circadian clock in the brain to peripheral clocks in bone [75]. Understanding the links between the circadian clock and bone resorption could advance diagnostics in periodontitis patients as well as reveal new targets for therapeutics.

Light therapy has been shown to be successful in wound healing in vivo, but until now no reason has been found for the mechanism behind these effects [76]. Now results show that *PER2* gene expression follows a circadian rhythm in human oral mucosa samples which is enhanced upon blue light exposure (460 nm) while no stimulation was achieved upon green light exposure (550 nm) [77]. Salivary glands form an important part of healthy oral mucosa and they were suggested to have a peripheral circadian clock. Besides clock components also the aqua channel aquaporin 5 (*Agp5*) displayed an oscillatory pattern under light-dark and dark-dark conditions. Additionally, it was shown that overexpression of Bmal1 leads to increased expression levels of *Agp5* [78]. Further, immunoglobulin A secretion was shown to be produced in a clock-dependent manner [79]. In attempts to set up a characterization of the human chronobiome, analysis of metabolites and microbiome of saliva were included. Saliva cortisol e.g. showed daily variations with morning peaks [80].

As light therapy was suggested for wound healing of soft tissues [76, 81] circadian clock rhythms could be assessed for their ability to stimulate oral soft tissue healing and periodontal regeneration in future therapeutic applications.

### Orthodontics

#### Biological aspects

Osteocalcin is part of the extracellular matrix in bone and produced by osteoblasts, thus it is regarded as marker for bone formation. It was reported that local and systemic production of osteocalcin increases when orthopedic force is applied on the mandible for 24 h daily compared to a 12 h application per day. One study looking deeper at molecular levels found that osteocalcin promoter activity is regulated in an oscillatory manner. This finding was most stably seen in the maxillomandibular complex. The study also confirmed that bone remodeling is accelerated in the resting phase [82]. Besides osteoblasts also osteoclasts are highly involved in bone remodeling. *Bmal1*, *Cry2* and *Per2* influence bone mass and bone volume via regulation of osteoclast parameters and differentiation [37, 38]. These phenotypes were found in knockout mice where BMAL1 in osteoclasts acts via the steroid receptor co-activator family and binds to the nuclear factor of activated T cells 1 promoter [37]. The acting mechanisms of *Cry2* and *Per2* remain unclarified. Deepening the understanding of the circadian clock mechanism in bone remodeling could open new possibilities for orthodontic treatment.

#### Clinical aspects

Successful orthodontic movement depends on the right choice of force and duration of the application. These orthodontic and orthopedic forces seem to vary in a circadian manner in its effects on bone remodeling in the maxillomandibular complex. According to these findings it can be suggested to adjust periods of wearing removable orthodontic appliances in resting phase, supporting a stronger effect and shortening wearing periods for patients. Pain perception and effectiveness of analgesic treatment were found to be associated with specific times of a day in bone disorders [83]. It is not known yet if orthodontic pain trajectories can also be correlated with circadian phases, but would be definitely of interest for improving orthodontic treatments.

## Discussion

**Fig. 3:**
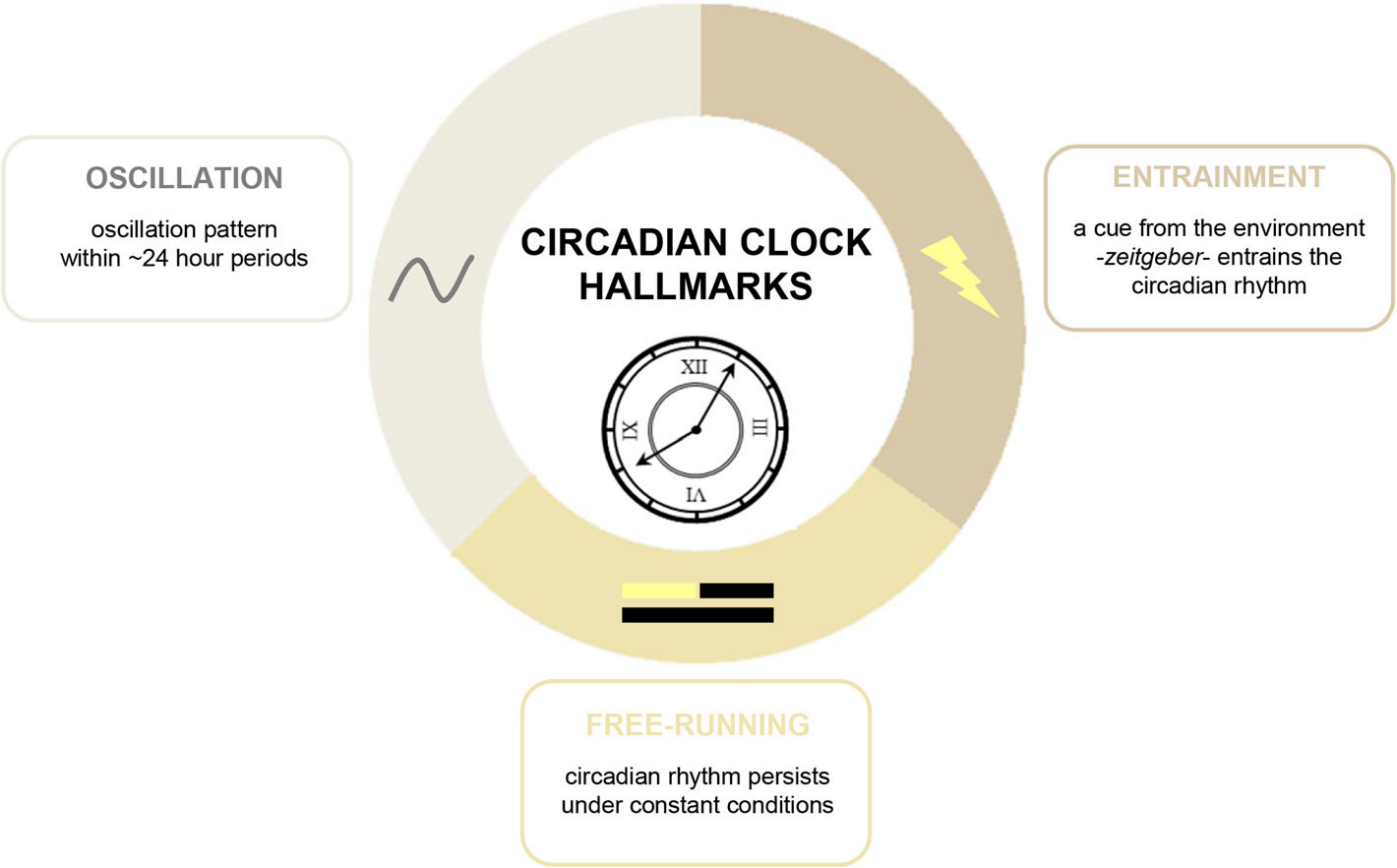
Circadian clock hallmarks. A functional circadian clock has to include three characteristics: gene or protein production has to follow an oscillation pattern over approximately 24 h, the circadian rhythm is entrained by an environmental stimulus (*zeitgeber* [official technical term]) and continues with its oscillation pattern, even after taking away the stimulus

The basis for chronodentistry has been set by demonstrating that dental pulp [84], periodontal tissues [11], oral mucosa [44], enamel [50], dentin [85] and mandibular bone [36] show clear evidence for the presence of peripheral clocks by producing its core components (Table 1). Specific functions of respective peripheral clocks in oral tissues and the mechanisms that are implied on the way to the fulfillment of such a function or behavior are still widely unknown. Meanwhile a dysfunctional clock mechanism has been attributed to be involved in the development of oral cancer [31–34] and juvenile skeletal mandibular hypoplasia [36]. Another step to put the pieces together is to find clock-controlled genes. A number of genes involved in oral biology were suggested to be produced in a circadian rhythm, but be careful when indicating a circadian pattern behind the production of genes or proteins. Except for so-called housekeeping genes and proteins, most gene expressions will show variances in production levels over the day, but this is by far not enough evidence to claim a circadian clock mechanism behind it. One of the most important characteristics of a circadian clock mechanism is that there is no action-reaction process, but a training process. Thus, entrainment status has to be assessed in free-running experiments, where the external stimulus is put away after a certain training period and the oscillation pattern still would be present. Taken together, a circadian clock mechanism has to be based on a repeating oscillation pattern, an entrainment by an environmental cue and free-running experiments (Fig. 3). Significant results should be supported not only by training, but also synchronization and determining oscillation patterns as well as cell cycle stages before and after training and synchronization.

**Table 1:** Circadian clock findings in dentistry. Major findings in chronobiology connected to oral and maxillofacial surgery, restorative dentistry, endodontics, periodontics and orthodontics are listed here

Field of dentistry	Molecule/Target	Major finding	Study model	Species	Reference
Oral & maxillofacial surgery	Clock, Bmal1, Tim, Cry1, Per1	oscillation in oral mucosa	in vitro/clinical	human	Bjarnason GA et al. (2001)
	Bmal1, Cry1, Cry2, Per1, Per2, Per3, Ck1ε	dysregulation is associated with tumor development stage in head and neck squamous cell carcinoma	in vitro/in vivo	human/mouse	Matsumoto CS et al. (2016)
	Per1, Per2, Dec1, Dec2, Cry1, Cry2, Npas2, Per3, Tim, Rorα, Rev-erbα	modulation is associated with cell proliferation, apoptosis and cell cycle progression in oral squamous cell carcinoma	in vitro	human	Wang Q et al. (2016), Zhao Q et al. (2016), Li H-X et al. (2016), Fu X-J et al. (2016)
	Per, Tim	dysregulation is associated with tumor size, invasion and patient survival	in vitro	human	Hsu C-M et al. (2012)
	Per1	association with tumor progression	in vitro	human	Chen R et al. (2012)
	Per1	association with carcinogenesis	in vivo	hamster	Ye H et al. (2015)
	Per1	association with later cancer stages and lymph node metastasis	in vitro/in vivo	human/mouse	Zhao N et al. (2013)
	Per1	modulation by titanium in bone marrow stromal cells	in vitro	human/rat/ mouse	Hassan N et al. (2017)
	Per1, Clock	recovery after surgery in head an neck squamous cell carcinoma patients with good prognosis	clinical	human	Hsu C-M et al. (2014)
	Per2	potential tumor suppressor	in vivo	hamster	Tan X-M et al. (2015)
	Per, Bmal1	association with tumor suppressor PTEN activity	in vitro/in vivo	human/mouse	Matsumoto CS et al. (2016)
	Bmal1	dysfunction is associated with juvenile skeletal mandibular hypoplasia	in vitro/in vivo	human/mouse	Zhao J et al. (2018)
	Tumor supressor genes Oncogenes	clock-controled genes in oral mucosa	in vitro	human	Zieker D et al. (2010)
Restorative dentistry	Clock, Bmal1, Per1, Per2	production in ameloblasts	in vitro/in vivo	rat/mouse	Zheng L et al. (2013)
	Clock, Bmal1, Per1, Per2	varying production during tooth development	in vivo	mouse	Zheng L et al. (2011)
	Per2	production in murine odontoblasts	in vivo	rat	Ohtsuka-Isoya M et al. (2001)
	Bmal1	stimulation of Amelx and Klk4stimulation of Amelx and Klk4	in vitro	rat	Zheng L et al. (2013)
	Bmal1	overexpression is associated with enamel morphology, thickness and hardness	in vitro	rat	Zheng L et al. (2013)
	Amelx, Lamp1, Slc4a4, Car2	light period-dependent production	in vitro/in vivo	mouse/rat	Lacruz RS et al. (2012)
	Ameloblast-specific genes Runx2	rhythmical production is associated with cell synchronization	in vitro	rat	Athanassiou-Papaefthymiou M et al. (2011)
	Collagen	production follows a circadian rhythm and might contributes to the rhythmicity of incremental lines in dentin	in vivo	rat	Ohtsuka M *et al.* (1998)
	Chronochemotherapy/ Chronoradiotherapy	reduction of adverse effects and stomatitis, improvement of treatment tolerance an survival time in oral squamous cell carcinoma and nasopharyngeal carcinoma	in vivo, clinical	mouse, human, mouse	Yang K et al. (2013), Zhang PX et al. (2018), Lin HX et al. (2013), Zhang Y et al. (2013)
	cross-striations and incremental line	potential correlationswith circadian periodicity	in vivo, *post mortem*	mouse, human, monkey	Sehic A et al. (2013), Antoine D et al. (2009), Smith TM et al. (2006)
Endodontics	Clock, Bmal1, Per1, Per2	sporadical production in dental pulp cells	in vivo	mouse	Zheng L et al. (2011)
	Clock, Bmal1, Per1, Per2, Per3, Cry1, Cry2	production by dental pulp-derived cells and modulation by hypoxic conditions	in vitro	human	Janjić K et al. (2018)
	Bmal1, Per2, Rev-erbα Bmal1, Per2, Rev-erbα	mechanical stretching can synchronize clock components in dental pulp stem cells	in vitro	human	Rogers EH et al. (2017)
	Per2, Per3	downregulation in dental pulps from carious teeth	in vitro	human	McLachlan JL et al. (2005)
	pulp sensibility	might follow diurnal rhythms	clinical	human	Guo B et al. (2007)
	pain sensation	possible correlation with circadian phases in oral region	clinical	human	Pöllmann L et al. (1987), Pöllmann L et al. (1978), Lemmer B et al. (1989), Lemmer B et al. (1991)
Periodontics	Clock, Bmal1, Per1, Per2, Per3, Cry1, Cry2	production in fibroblasts from gingiva and periodontal ligament and modulation by hypoxic conditions	in vitro	human	Janjić K et al. (2017)
	Bmal1, Clock, Per1, Per2	produced in oral mucosa	in vitro/in vivo	human/mouse	Zheng L et al. (2012)
	Clock:Bmal1 dimer	stimulation of SMAD3 promotor activity in gingival fibroblasts	in vitro/in vivo	human/mouse	Sato F et al. (2012)
	Bmal1	increase of Agp5	in vitro/in vivo	human/mouse	Zheng L et al. (2012)
	IL-1β	production in diurnal rhythms in crevicular fluid	clinical	human	Bergmann A et al. (2008)
	periodontal indices	FMBS, FMPS, PSR and PRA show diurnal variations	clinical	human	Bertoldi C et al. (2017)
Orthodontics	Osteocalcin	promotor activity is regulated in an oscillatory manner in the maxillomandibular complex	in vivo	mouse	Gafni Y et al. (2009)

Chronodentistry is still some steps away from patient application, but already shows potential in the different fields of dentistry including restorative dentistry, endodontics, periodontics, orthodontics and oral and maxillofacial surgery. The field of pediatric dentistry has not been covered yet on any aspect of the circadian clock. Although, it would be interesting if genes that are involved in tooth development, change from deciduous to permanent dentition or development of oral pediatric disorders are clock-controlled and display circadian behavior. Particularly for children, non-invasive treatment approaches as light therapy are desirable. Light being the strongest known *zeitgeber* could easily be used as therapeutic tool and could be optimized for photodynamic therapies in endodontics or light therapy for wound healing of periodontal tissues and oral mucosa. However, for this purpose the role of the circadian clock in the oral tissues needs to be understood. Besides new therapeutic approaches, assessment of patient chronotypes could improve treatment and diagnosis timings to save time, money and drug overload for the patient, altogether making medication more efficient. Recently the relation between a specific chronotype and bruxism was discussed, since bruxism can be assigned to different times of day. First results of clinical studies rather show a tendency to no correlation between chronotypes and bruxism [86, 87].

Circadian clocks are only one type of molecular clocks. There are also clock mechanisms for longer period intervals as for example circalunar clocks. These molecular clocks with longer time frames might be of interest for chronic diseases or diseases with a long time of development as cancer. These clocks are generally barely studied and completely unidentified in oral biology or dentistry. Further there are indications that molecular clocks play a role in aging [88] and forensics [89, 90]. These specialties are not directly related with clinical dentistry, although could be interesting for the broad field of oral medicine.

## Conclusion

The field of chronodentistry has been founded on a solid and promising basis to revolutionize dentistry with new or refined therapeutic approaches and to bring light into basic mechanisms of oral biology.

## Appendix

### Literature selection criteria

Literature used in the main part of this review was selected after a PubMed (https://www.ncbi.nlm.nih.gov/pubmed) search using the search terms “dentistry circadian clock”, “tooth circadian clock”, “oral surgery circadian clock”, “maxillofacial circadian clock”, “enamel circadian clock”, “dentin circadian clock”, “dental pulp circadian clock”, “endodontics circadian clock”, “endodontology circadian clock”, “periodontics circadian clock”, “periodontology circadian clock”, “gingiva circadian clock”, “periodontal ligament circadian clock”, “orthodontics circadian clock” an “bone circadian clock”. All literature containing findings on the circadian clock in relation to dentistry or dental tissues was selected for the main part of this review.

## Abbreviations

Agp5: Aquaporin 5
Arntl or Bmal1: Aryl hydrocarbon receptor nuclear translocator-like
CK1ε: Casein kinase 1ε
Clock: Circadian locomotor output cycles kaput (
Cry1–2: Cryptochrome 1–2
DEC1–2: Differentiated embryo-chondrocyte 1–2
Dlx3: Distal-less homeobox 3
Enam: Enamelin
FMBS: Full-mouth bleeding score
FMPS: Full-mouth plaque score
HIF-1: Hypoxia-inducible factor-1
IL: Interleukin
Per1–3: Period 1–3
PRA: Periodontal risk assessment
PSR: Periodontal screening and recording
ROS: Reactive oxygen species
Runx2: Runt-related transcription factor 2
TIM: Timeless
